# Chemicals from textiles to skin: an in vitro permeation study of benzothiazole

**DOI:** 10.1007/s11356-018-2448-6

**Published:** 2018-06-17

**Authors:** Francesco Iadaresta, Michele Dario Manniello, Conny Östman, Carlo Crescenzi, Jan Holmbäck, Paola Russo

**Affiliations:** 1 Department of Environmental Sciences and Analytical Chemistry (ACES), Stockholm University, Arrhenius Laboratory, Stockholm University, SE-10691 Stockholm, Sweden; 2 Department of Pharmacy, University of Salerno, Via Giovanni Paolo II, 132, I-84084 Fisciano, SA Italy

**Keywords:** Benzothiazole, Permeation study, Textiles, Clothes, Franz cell, Flow-through diffusion cell, Strat-M®, Dermal exposure, Risk assessment

## Abstract

Despite the possible impact on human health, few studies have been conducted to assess the penetration and accumulation of contaminants in the skin after a prolonged contact with textile materials. In previous studies, we have shown that benzothiazole and its derivatives, as well as other potentially hazardous chemicals, often are present as textile contaminants in clothes available on the retail market. Since benzothiazole is a common contaminant in clothes, these can be a possible route for human chemical exposure, both systemic and onto the skin. To investigate this potential exposure, Franz-type and flow-through cells were used for the permeation studies together with a Strat-M® artificial membranes. Experiments were performed using solutions of benzothiazole, as well as contaminated textile samples in the donor chamber. Benzothiazole was demonstrated to penetrate through, as well as being accumulated in the membrane mimicking the skin. After 24 h, up to 62% of benzothiazole was found in the acceptor cell, while up to 37% was found absorbed in the skin mimicking membrane. It also was shown that there was release and permeation from contaminated fabrics. The results indicate that benzothiazole can be released from textile materials, penetrate through the skin, and further enter the human body. This will possibly also apply to other chemical contaminants in textiles, and the results of this study indicate that the presence of these textile contaminants entails potential health risks. A rough risk assessment was made for clothing textiles according to Environmental Protection Agency (EPA) and European regulations for carcinogenic and non-carcinogenic compounds, using literature data for benzothiazole.

**Electronic supplementary material:**

The online version of this article (10.1007/s11356-018-2448-6) contains supplementary material, which is available to authorized users.

## Introduction

The skin protects the human body from water loss, harmful microorganisms, irritants, and injuries, and is an important barrier against exposure to environmental contaminants. Clothes provide an additional barrier, but the direct and prolonged interaction between the *stratum corneum* and the textile material, the latter often synthetic, may be responsible for irritation, sensitization, or even penetration of hazardous chemicals into the human body (Zhong 2006).

Chemicals present in textiles have various sources. Chemical products can be added for specific purposes during the manufacturing process, or involuntarily added as by-products. One of the main chemical sources in textile production is the coloring step in which the dyes are added (KemI 2014). Depending on the quality of the chemicals used for the coloring, as well as on the coloring/washing procedures, the remaining chemical impurities of the dye’s formulation present in the textile may constitute a health risk if taken up by and passing through the human skin. In vitro mutagenicity and cytotoxicity studies have been conducted to assess the risk of chronic exposure to fabric dyes (de Aragão Umbuzeiro 2005; Tsuboy 2007; Ferraz 2012). In the production step of natural fabrics (e.g., cotton), several pesticides are often used during storage (Thompson 2015; Zhang 2008), or to improve the technical characteristics of the final product, obtaining an anti-bug cloth (Holme 2007). It has also been shown that generation of toxic intermediates of chemicals used in textiles can occur, e.g., azo-reduction by anaerobic bacteria in the skin, as well as other kinds of degradation (Cervantes 2011; Hunger et al., 2002; Spadaro 1994).

There are few studies conducted assessing the skin absorption and penetration of chemicals present in textile materials in contact with the skin (BFR 2012). In the past, an in vivo experiment has shown that a phosphorous flame retardant (PFR) impregnated in children’s sleepwear was able to migrate from the fabric to the skin and further into the human body, and its metabolite was detected in the subject’s urine (Blum 1978). Current knowledge of PFR accumulation and the consequences on the human health is however still incomplete and additional studies are needed (Tajima et al. 2014). However, recent pharmacokinetic studies point out that the dermal contact is an important pathway of human chemical exposure to brominated flame retardants (Abdallah 2015).

Benzothiazole (BT) and its derivatives have several industrial applications and are frequently used as, e.g., cyanine dyes, biocides, herbicides, and fungicides (Kloepfer 2004). They are also used as precursor in the synthesis of vulcanization accelerators in rubber production (e.g., 2-morpholinothiobenzothiazole) and as corrosion inhibitors (e.g., 2-mercaptobenzothiazole in paper production) (Milanova 2001). BT has a meaty, nutty, or coffee taste and it is added in foods as a flavoring agent at 0.5 ppm National Toxicology Program (NTP) (n.d.). Intravenous, intraperitoneal, and dermal LD_50_ range from 95 to 200 mg/kg, and the acute toxicity of BT is characterized by a depression of the central nervous and respiratory systems as well as kidney and liver toxicity (Ginsberg 2011). BT can be considered as a skin allergen since a positive dermatitis reaction occurred in 17 of 43 human subjects treated topically (Bogert 1932). In previous works, we have shown that a number of potentially hazardous chemicals, among these BT and its derivatives, are frequently present in common textile materials (Luongo G 2014, Avagyan R 2015, Luongo et al., 2016a, b, Avagyan R 2013). The presence of BT in textiles has also been confirmed by other researchers (Liu 2017). We have also shown that the concentration of BT decreased in the fabrics when washed, demonstrating a mobility of this chemical from the fabrics (Luongo et al. 2016a). Added together, these results point out a need of appropriate in vitro studies to investigate the release of BT from textile materials and determine if it migrates to, is absorbed by, and/or permeate through the skin.

When designing in vitro permeation experiments, the selection of the membrane is important since it affects the prediction of the compound’s penetration. Different types of membranes, such as inert artificial membranes (e.g., polytetrafluoroethylene), real human skin, animal tissues (Fabrizio et al. 2009), or human skin equivalent (HSE) models (Eilstein 2014), can be used depending on the purpose of the study. Several of these membranes suffer from batch-to-batch variability, poor stability, and sensitivity to storage. HSEs, such as reconstructed human epidermis (RHE) or full-thickness skin (FT), are membranes made by cell layers that are able to mimic the human skin tissue (Abdallah 2015).

The Strat-M® Transdermal Diffusion Membrane is an artificial, non-animal based, human skin mimicking synthetic membrane used in many permeation studies. It simulates the different layers of the human skin (the *stratum corneum*, the epidermis and the dermis), and has shown good correlation with human cadaver skin for several compounds (e.g.*,* caffeine, nicotine) (Strat-M® Transdermal Diffusion Membrane, Uchida 2015).

The aim of this study was to predict the permeation through and accumulation in the skin of BT, investigate the release of BT from textile materials and to use the data obtained from these in vitro permeation studies to make a rough estimate of the skin exposure of BT and the correlated risk for human health.

## Materials and methods

### Chemicals and solvents

All solvents used were of HPLC grade. Methanol, dichloromethane, water, and formic acid were purchased from VWR International (Spånga, Sweden), while acetonitrile was purchased from Rathburn Chemicals (Walkerburn, Scotland). Benzothiazole (BT), CAS No. 95-16-9, 2-methyl benzothiazole (MeBT) CAS No. 120-75-2, and chemicals used for the permeation studies (KH_2_PO_4_, NaOH) were supplied by Sigma-Aldrich (Merck KGaA, Darmstadt, Germany). The syringe filters had a pore size of 0.45 μm, and a diameter 4 mm (NTK Kemi, Uppsala, Sweden), and Strat-M® membranes were purchased from Merck Millipore (Billerica, MA, USA).

### Instrumental analysis

#### LC/MS/MS system

The LC/MS/MS instrument consisted of an Agilent high-performance liquid chromatography (HPLC) system with a 1260 binary pump, a 1100 degasser, and a 1100 autosampler (Wilmington, DE, USA) coupled to an API QTrap™ triple quadrupole mass spectrometer (PE Sciex, Toronto, ON, Canada) equipped with a TurboIon® electrospray interface operating in positive ion mode. The LC/MS/MS method has been described previously (Luongo et al., 2016a, b). Briefly, the chromatographic separation was performed using an ACE 3 C8 microbore column (l = 50 mm, i.d. = 2.1 mm, *d*_p_ = 3 μm) equipped with a ACE 3 C8 guard column (l = 10 mm, i.d. = 2.1 mm, dp = 3 μm), both manufactured by Advanced Chromatography Technologies (Aberdeen, Scotland). Mobile phase A consisted of 0.1% (*v*:*v*) formic acid in pure water and mobile phase B 0.1% (*v*:*v*) formic acid in acetonitrile. The flowrate was set to 200 μL/min with mobile phase consisting of 35% B as the initial conditions. Between 2 and 5 min, a linear gradient from 35 to 95% B was applied and the final conditions were kept for 25 min after which the system was reset to initial conditions. An equilibration time of 10 min at 35% mobile phase B was kept before each run. The injection volume was 5 μL. The mass spectrometer (MS) was operated in selected reaction monitoring (SRM) mode with settings shown in Table S1 in the electronic supplementary material (ESM).

#### LC/UV system

An Agilent HPLC system with a 1100 binary pump solvent delivery system, a 1100 degasser, and a 1100 autosampler (Wilmington, DE, USA) coupled to a 1100 Agilent diode array detector (DAD) with the detection wavelength set to 230 nm was used as LC/UV system. Chromatographic separation was performed using an Aquasil C18 column (l = 50 mm, i.d. = 4.6 mm, dp = 3 μm, Keystone Scientific Inc., Bellefonte, PA, USA). The mobile phases had the same composition as for the LC/MS/MS system. In this case, the flowrate was set to 1 mL/min and the injection volume was 10 μL. The gradient started at 25% B which was held for 0.2 min before increasing to 50% B during 2.3 min, and further to 80% B during 2.4 min. Eighty percent B was held for 1 min before increasing to 95% B which was held for 2 min after which the system was set back to initial conditions. The column was conditioned for 2 min with 25% B before next run. Evaluation method data are presented in the ESM.

### Franz cell experimental conditions

**Fig 1.**
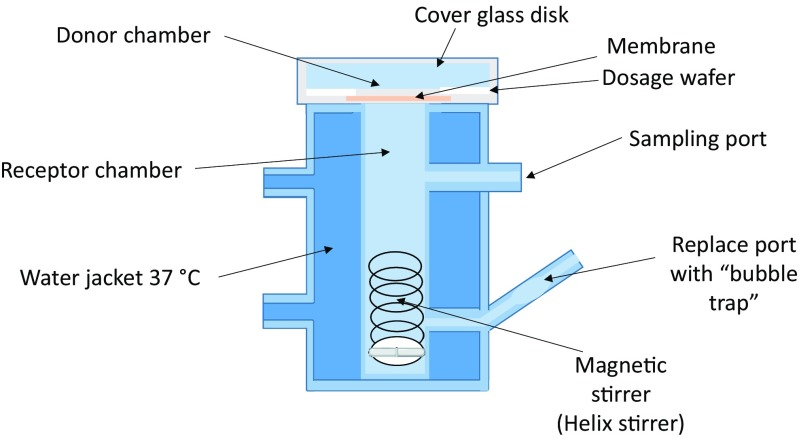
Schematic of a vertical Franz-type diffusion cell.

In a first set of experiments, the ability of BT to interact with and penetrate through the Strat-M® membrane was evaluated using a Franz-type vertical diffusion cell, Fig. 1 (Hanson Teledyne Research Corporation, Chatsworth, CA, USA). This type of diffusion cell uses small volumes (i.e., 4 to 7 mL) of solvent in the receiving compartment (Russo 2013).

During the permeation experiments, the BT water solubility (4.3 mg/mL) (Pubchem 2017) ensures sink conditions even with the small volumes involved. The experiment was performed as reported elsewhere (Stigliani 2016). Briefly, the Strat-M® membrane was placed between the donor and receiving chambers. The receptor chamber (7 mL) was filled with a 50-mM phosphate buffer saline (PBS) at pH adjusted to 6.8 with NaOH 2 N, and kept under continuous magnetic stirring (170 rpm), with the system temperature set to 37.2 °C, and using an initial equilibration time of 30 min. The donor cell was then filled with 300 μl of 1 mg/mL BT in water making sure that the donor solution was covering the entire permeation area (1.77 cm^2^). The amount of BT added to the donor compartment was selected by taking into account both the LOQ of the analytical method (~ 110 pg injected) at first withdrawal, as well as the required sink condition throughout the permeation study. The experiment was run for 24 h during which eight withdrawals of 250 μL were made from the receiving solution at 30, 60, 120, 180, 240, 300, 360, and 1440 min, respectively. After each withdrawal, the receiving solution was refilled by injecting 250 μL of the same PBS buffer solution kept at 37.2 °C. Prior to the LC/MS/MS analysis, 100 μl of methylbenzothiazole (MeBT) (10 μg/mL in methanol) was added as internal standard to each of the withdrawn receiving solutions, as well as to the membrane and to the donor solution prior to extraction. The mass balance expressed as BT recovery was calculated from the ratio between the total quantified amount (i.e., the sum of the amounts of BT extracted from the membrane, total amount of BT permeated into the acceptor solution during 24 h, and BT extracted from the donor solution) and the nominal spiked amount.

### Flow-through diffusion cell experimental conditions

**Fig. 2.**
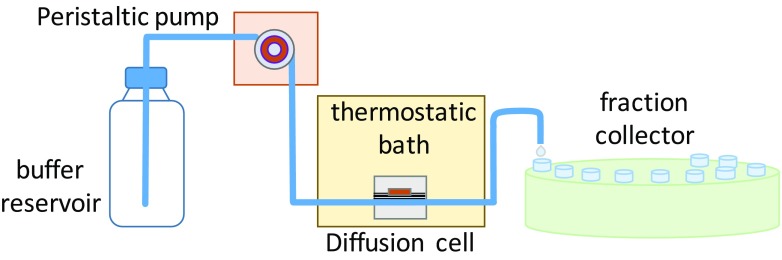
Schematic of the flow-through diffusion cell system.

A description of the flow-through diffusion cells used in this work, Fig. 2, has been reported previously (Lodén 2004). Briefly, the system consisted of a buffer reservoir containing 11.8 mM PBS at pH 7.4 (saline concentration for Na^+^ at 157 mM, K^+^ at 4.5 mM, Cl^−^ at 139 mM), an eight-channel peristaltic pump, eight flow-throw diffusion cells with 0.5 cm^2^ cross section placed on a stainless steel platform kept at 37 °C, and an eight-channel fraction collector. To minimize bubble formation, the buffer solution was degassed by vacuum-assisted ultra-sonication prior to use, and transported into the system using Teflon tubing (0.5 mm ID). A Strat-M® membrane pre-equilibrated for 30 min in buffer solution was placed between the donor and the receiving chamber, and the flow rate adjusted to approximately 1.5 mL/h. The donor chamber was covered with aluminum foil to shield from light and avoid contamination and solvent evaporation. Seven fractions of the receptor fluid were collected during 0–2, 2–4, 4–6, 6–10, 10–14, 14–18, and 18–24 h, respectively.

When investigating permeation from water, 85 μL of BT solution (1.4 mg/mL in water) was used as donor phase. In the experiments with spiked textile pieces, the fabric was placed on the top of the membrane in the donor chamber covering the cross section (0.5 cm^2^), and spiked with 50 μL of BT solution (0.228 mg/mL in H_2_O:EtOH, 50:50 *v*:*v*). The volume of each collected fraction was calculated from its weight and an aliquot of the fraction was analyzed. These experiments were performed in triplicates and the analyses performed by LC/UV. After collecting the last fraction, both the membrane and the textile pieces were extracted by ultra-sonication in 1 mL of methanol, see below, and analyzed with the same method. The mass balance expressed as BT recovery was calculated from the ratio between the total quantified amount (i.e., the sum of BT permeated during 24 h, BT extracted from the spiked cloth, and BT extracted from the membrane) and the nominal spiked amount where the quantifications were performed by external calibration.

### Membrane extraction

In the Franz cell experiments, the membranes were spiked with 100 μL of internal standard solution before the extraction procedure and left for 30 min to let the solvent evaporate. The membranes were subsequently extracted twice with 6 mL of dichloromethane:MeOH 2:1 (*v*:*v*) assisted by ultra-sonication for 10 min (Avagyan 2015). Before evaporation, the extract was filtered and 0.5 mL water was added as a keeper. The extract was solvent reduced under a stream of nitrogen at 45 °C down to approximatively 0.5 mL, to which an additional 0.5 mL of methanol was added.

In the flow-through diffusion cell experiments, the membranes and the cloths were extracted using 1 mL of methanol assisted by ultra-sonication for 5 min, left in methanol over the night (~ 16 h), ultra-sonicated for an additional 5 min, and finally filtered prior to analysis with LC/UV.

### Risk assessment evaluation

Dermal exposure was calculated using Eq. 1 which is based on the European Chemical Agency guidance (ECHA 2012) and suggested by Rovira et al. (Rovira 2015):

1 $$ \mathrm{Exp}\ \mathrm{derm}=\mathrm{Conc}\ \mathrm{cloth}\ast \mathrm{d}\ \mathrm{cloth}\ast \mathrm{A}\ \mathrm{skin}\ast \mathrm{F}\ \mathrm{contact}\ast \kern0.5em \mathrm{F}\ \mathrm{mig}\kern0.5em \ast \kern0.5em \mathrm{F}\ \mathrm{pen}\kern0.5em \ast \mathrm{T}\ \mathrm{contact}\kern0.5em \ast \frac{\mathrm{n}}{\mathrm{BW}} $$

The equation includes the concentration of the chemical in the cloth (Conc cloth in g/g), cloth areal density (d cloth in mg/cm^2^), body contact surface (A skin ∗ F contact in cm^2^, where A skin is the total textile skin contact area, and F contact is the fraction of the textile area in actual contact with the skin), contact time (T contact in days), migration rate (F mig in days^−1^), permeation rate (F pen unitless), wear events per day (n in day^−1^), and body weight (BW in kg).

In the dermal exposure calculations, a t-shirt was used as the model textile, with the selected exposure parameters shown in Table 1. The BT concentration (Conc_cloth_) was set to the median concentration in eight different t-shirts determined by Luongo et al. (Luongo 2016a), Table S2 in ESM, and the cloth areal density (d cloth) was set as the average of seven different t-shirts from data reported by Rovira et al. (Rovira 2015), Table S3 in ESM.). The average skin area for a t-shirt and the body weight values was 7030 cm^2^ and 65 kg, respectively (U.S. EPA 2011). The fraction of contact area for the skin (F contact) was set to 1, as for a tight t-shirt, and the duration of contact between skin and textile was set to 16 h (0.67 day). The skin penetration factor (F pen) was experimentally obtained from vertical Franz cell and flow-through diffusion cell from water solutions and spiked cloths. The carcinogenic risk was evaluated using the slope factor (SF) 6.34E-07/μg kg^−1^ day^−1^ calculated for 2-mercaptobenzothiazole (2-MBZT) (Ginsberg 2011).

**Table 1:** Parameters used in the risk assessment estimation by Eq. 1

Parameter	Description	Unit	Value	Reference
Conc_cloth_	T-shirt BT median concentration	g/g	4.0 × 10^−4^	Luongo et al., 2016a, b
*d*_cloth_	T-shirt mean areal density	mg/cm^2^	16.3	Rovira 2015
*A*_skin_	Mean exposed skin area (adult)	cm^2^	7030.5	US EPA 2011
*F*_contact_	Fraction of skin contact area	Unitless	1	*Tight t-shirt*
*F*_mig_	Fraction of substance migrating to skin^1)^	%	0.1	BfR 2012
*F*_pen_	Fraction of penetration inside body^1)^	%	50	U.S. EPA O, 2014, b
*T*_contact_	Contact duration between skin-textile	day	0.67	BfR 2012
*n*	Mean number of events per day	day^−1^	1	*Assumed*
BW	Body weight^2)^	kg	65	US EPA 2011
	First wear (hours)	hours	16	*Assumed*
	Exposed skin area, adult male (T-shirt)	cm^2^	7120	US EPA 2011
	Exposed skin area, adult female (Blouse)	cm^2^	6941	US EPA 2011
	Exposed skin area, child < 1 year (Pajamas)^3)^	cm^2^	2754	US EPA 2011
	Exposed skin area, child < 1 year (Bodysuit)^3)^	cm^2^	1285	US EPA 2011

^1)^Default values for hydrophobic textile auxiliary

^2)^ Average weight of adult male and female (70 and 60 kg, respectively)

^3)^Average exposed area of pajamas and bodysuit

### First-order kinetic model fitting

To calculate the percentage of compound permeated after 16 h first wearing, the penetration profiles obtained from the experiments were fitted to a first order kinetic model according to Fick’s law of diffusion, as expressed in Eq. 2:

2 $$ \frac{m_{rec}}{m_0}=1-{e}^{- kt} $$

where *m*_*rec*_ is the accumulated mass of BT in the receiving fluid, *m*_0_ is the total amount penetrated after infinite time (asymptotic value which cannot be determined experimentally), *k* is the rate constant, and *t* is the time in hours. Equation 2 can be rewritten as

3 $$ \ln \left(1-\frac{m_{rec}}{m_0}\right)=- kt $$

Equation 3 was used to estimate *m*_0_ and *k* by least squares fitting using Solver in Microsoft Excel. Briefly, *k* is calculated from the experimental data starting from a hypothetical *m*_0_, selected slightly higher than the experimental value at 24 h. The sum of squares of the errors for the experimental values fitted to the equation was calculated and minimized by changing *m*_0_.

## Results and discussion

### Permeation of benzothiazole from a water solution and spiked cloths

The selection of BT as the model compound for this study was due to its frequent occurrence in clothing materials and its water solubility. In permeation studies, the water solubility of the investigated compound is an important issue since it will affect the permeation process. BT is slightly soluble in water (4.3 mg/mL at 25 °C (Pubchem)) and throughout the experiments, the concentration was less than 10% of its solubility, ensuring the desired sink conditions. It should be pointed out that the aim of this study was to determine the transfer of BT from a t-shirt, as well as to determine skin accumulation and permeation rate of BT. Thus, the concentrations used in the experiments were adapted for multiple withdrawals of the studied compound in combination with the LOD of the applied analytical methods and not to simulate concentrations found in clothing textiles.

**Fig. 3.**
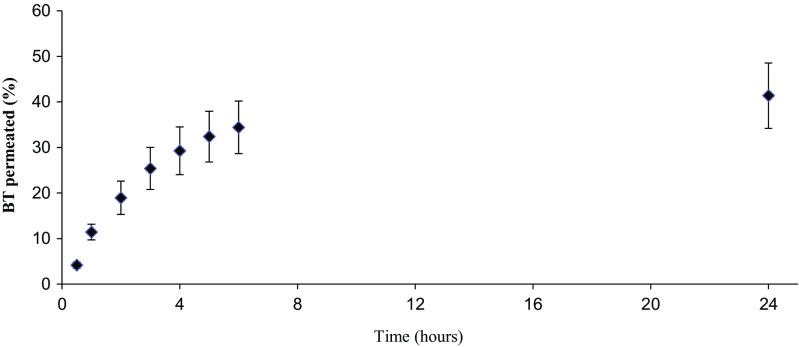
BT permeation curve trough the Strat-M membrane using a Franz cell (n=5).

To evaluate the BT permeation through the Strat-M® membrane, permeation assays were performed using Franz-type vertical diffusion cells. The data are reported as the percentage of BT transported through the membrane as a function of time, Fig. 3. By fitting the experimental data to a first-order kinetic model, as expressed in Eq. 3, the rate constant *k* was estimated to 0.303, and the total amount of permeated BT at infinite time (*m*_0_) to 41.4% of the spiked amount with a coefficient of determination (*R*^2^) of 0.9998. The total recovery of BT after 24 h was 80.4% ± 6.4% (*n* = 5, standard error), where 41.3% ± 5.9% was found in the receiving chamber, 37.9% ± 3.3% was absorbed in the membrane, and 1.2% ± 0.6% left in the donor solution.

**Fig. 4.**
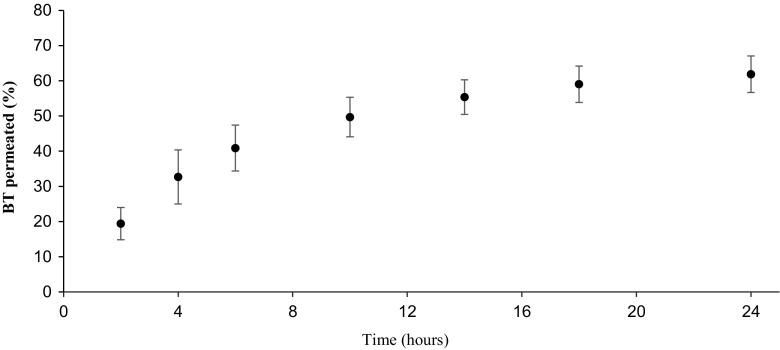
BT permeation curve trough the Strat-M membrane using a flow-through diffusion cell (n=4).

A flow-through diffusion cell system, described above in section *2.4*, was set up to be able to work with larger amounts of solvent and obtain a versatile method for evaluating the permeation of both hydrophilic as well as lipophilic molecules. Results obtained from water solutions of BT using the flow-through diffusion cell equipment are shown in Fig. 4. A least squares fit to Eq. 3 in this case estimates the rate constant *k* to 0.151 and *m*_0_ to 63.5% of spiked amount with an *R*^2^ of 0.996. The total recovery of BT after 24 h was 70.7% ± 3.9% (*n* = 4, standard error), distributed as 62.0% ± 5.2% in the receiving chamber and 8.6% ± 1.4% absorbed in the membrane, while the recovered amount of donor solution was insufficient for quantification.

**Fig. 5.**
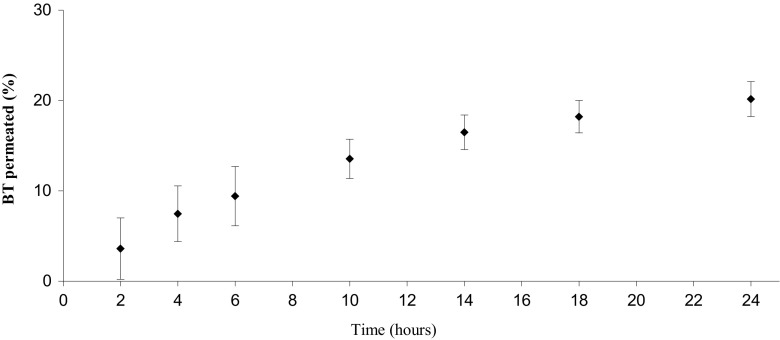
BT permeation curve from spiked cloths using a flow-through diffusion cell (n=3).

Permeation from textile materials was studied using the flow-through diffusion cell method to investigate if it was possible for BT to migrate from a spiked textile, permeate through the Strat-M® membrane and further into the receptor chamber. The obtained permeation curve is shown in Fig. 5. Fitting data to Eq. 3 estimates *k* to 0.091 and *m*_0_ to 30.2% with *R*^2^ of 0.9995. The total recovery after 24 h was 56.9% with standard error ± 3.2% (*n* = 3), distributed between 26.9% ± 1.9% in the receiving chamber, 16.7% ± 2.4% absorbed in the membrane, and 13.3% ± 4.6% remaining in the textile.

### Franz cells vs flow-through diffusion cells

The most common drawback in the in vitro models used here is the risk to get air into the system, which will impair the permeation through the membrane during the experiments. While the Franz cell is the standard method and meets the requirements in the pharmacopeia standards, the flow-through diffusion cells are more suitable for textile-related compounds since the receiving fluid is constantly replaced, which will keep the sink conditions throughout the experiment. The flow-through diffusion cell model also simulates the blood flow beneath the skin, since it has a continuous flow of the receptor fluid removing the permeated material from the receiving chamber. Further, by using a fraction collector, the sampling can be automated, which allows sample collection over the night, in contrast to the Franz cell where sampling has to be done manually and is thus inconvenient to perform during non-working hours.

Drawbacks of the flow-through diffusion cell system could be a possible adsorption in the tubing connections of the investigated compounds, limit of quantification issues due to dilution by the receptor fluid, and a possible loss due to evaporation of volatile compounds, the latter since the system’s collection step is open.

**Fig. 6.**
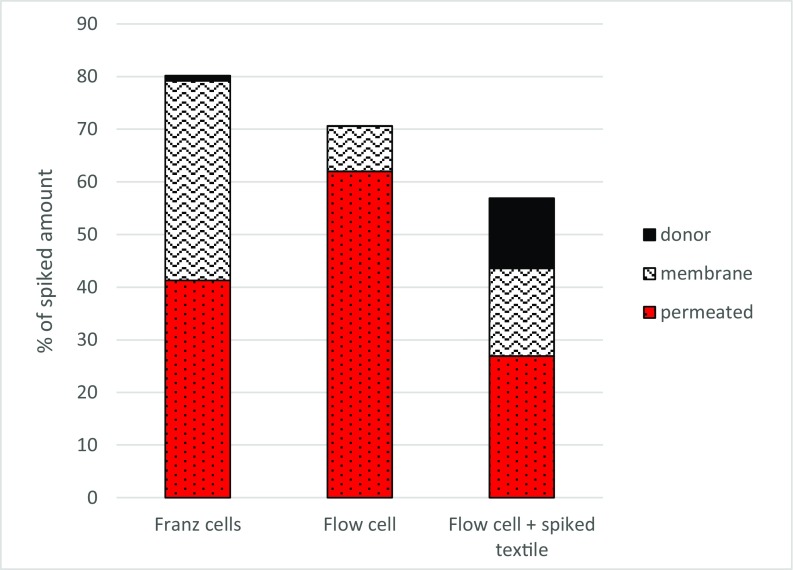
Mass balance of benzothiazole in the different compartment after 24 hours. The first two bars are for BT in water solution, the third for a BT spiked textile.

In Fig. 6, the mass balance for the three experiments is shown. The amount of BT permeated after 24 h in the flow-through diffusion cell experiments is higher than in the Franz cells, which most likely is due the larger total volume of receiving fluid. This dynamic system never reaches equilibrium, and due to the transport of permeated material away from the membrane, it most likely simulates the permeation through living skin in a better way.

### Dermal exposure assessment

The data from the permeation experiments are used below to estimate dermal exposure at first wear of a hypothetical new, unwashed t-shirt. The dermal exposure calculation (Eq. 1) is dependent on the cloth (the concentration of the compound in the textile and the density of the cloth), the skin contact of the cloth (skin area, contact time, contact fraction, body weight, and number of events per day) as well as of the properties of the compound. Two compound-depending factors must be experimentally calculated, migration rate and penetration factor. The migration rate expresses the compound’s mobility from the cloth to the membrane (*F*_mig_), while the penetration factor gives the percentage of absorption through the membrane (*F*_pen_). If these values are not available, it is possible to use tabulated values considering the worst case assumption (BfR 2012). These values are shown in Table 2. In this study, we estimate the penetration factor experimentally by means of either the flow-through diffusion cell or the Franz cell system.

**Table 2:** Worst case assumption if no experimental data are available (BFR 2012)

Substance class	Migration factor	Penetration factor
Dye	0.5%	1%*/**
Hydrophilic compound	2%	5%*
Hydrophobic textile auxiliary	0.1%	50%*

*Exception molecular weight > 700 or log Pow < − 1 or > 6

**2% is used in perspiration zone

The migration factor was determined experimentally using spiked cloths. However, these experiments take into account both the transfer of BT from the fabric to the membrane, as well as the permeation through the membrane. This will give a new factor in the equation, which includes both the migration factor and the penetration factor. The experiments with spiked clothing in the flow-through diffusion cell showed a lower rate constant and lower percentage of permeated BT compared to the water solution experiments in the Franz-type diffusion cell. However, the dermal exposure is probably overestimated since the amount of spiked BT required to get quantifiable concentrations and the limited piece of textile might result in an overload of the fabric. Further studies of experimental determination of migration factors are needed since many factors can affect the determination. Migration from different types of fabrics should be investigated, sweat surrogate should be used and a subsequent concentration step applied to the extract to make it quantifiable.

Data from the present study has been used to calculate the BT dermal exposure from a t-shirt using the median concentration from our previous studies, while the mean t-shirt density and the migration factor were taken from the literature (Table 2). All the values are specified in Table 1. The penetration was calculated assuming a first wear of 16 h, following the BFR suggestion, and using the value from the penetration curve fitted to the experimental data.

The dermal exposure calculated using the data from this study considering the lowest and the highest scenario gave a range between 0.54 and 1.36 μg/kg body weight.

There are limited toxicological data for BT in literature, but a rough estimate of the non-carcinogenic risk for the hypothetical t-shirt can be made by using the oral reference dose (RfD) specific to dietary exposure defined by US EPA (U.S. EPA O, 2014, b). This is similar to the acceptable daily intake (ADI) used by the Joint FAO/WHO Expert Committee on Food Additives. It is however necessary to consider the limitation of this risk assessment since the dermal exposure has been estimated using a simplified model. The RfD is derived from an experimental no-observed-adverse-effect (NOAEL) daily dose calculated according to Eq. 4 (U.S. EPA O, 2014, b):

4 $$ RfD=\frac{NOAEL}{Uf_{inter}\times {Uf}_{intra}\times {Uf}_{other}} $$

The Uf_inter_ and Uf_intra_ factors are inter and intra species variability uncertainty factors which both have the value 10 (U.S. EPA O, 2014, b). An extra modifying factor Uf_other_, also with the value 10, is used when few data are available (EPA 2017). For BT, there are no published NOAEL values, but a 90-day dietary study submitted to the World Health Organization (WHO) in which rats were dosed with 5.1 mg kg^−1^ day^−1^ of BT reports no toxic effect. If we use this value as the NOAEL for BT, the RfD for the BT is estimated to 5 μg kg^−1^ day^−1^, i.e., NOAEL/1000 (Ginsberg 2011). The highest value of daily intake for BT via skin obtained in this study was 1.36 μg kg^−1^ day^−1^ which is close to the calculated RfD.

The non-carcinogenic risk can then be expressed as the hazard quotient (HQ). This is obtained by dividing the calculated dermal exposure (1.36 μg kg^−1^ day^−1^) by the estimated RfD (5 μg kg^−1^ day^−1^). For the hypothetical t-shirt used for these calculations, the HQ can be estimated to 0.27 indicating an exposure below the acceptable daily intake BT according to US EPA 2014. However, it should be pointed out that the RfD is specific to dietary exposure, not exposure to the skin.

### Sensitization and carcinogenic risk

Even though BT is classified as a mild to moderate skin irritant (EPA 2017), it was not possible to calculate the sensitization risk from the clothing since there is no dose per unit area available in the literature. In our experiment, we showed that a significant percentage of BT was absorbed in the membrane and further studies are required to evaluate the possible topical effect generated by BT in the skin.

The carcinogenic risk is evaluated from the worst calculated dermal exposure (1.36E-03 mg kg^−1^ day^−1^). This value is multiplied by the slope factor, but since there are no carcinogenic studies for the BT, the value suggested in literature by (Ginsberg 2011) is the calculated slope factor for the 2-MBTZ (6.34E-04 mg kg^−1^ day^−1^). The resulting cancer risk is of 0.86 E-6, thus less than 1 in a million cancer risk level, considered to be within the acceptable range (< 10^−6^) according to the international standards (US EPA 2014b). However, this evaluation of carcinogenic risk has limitations: the used slope factor is calculated for 2-MBTZ and not the BT; it is calculated from an acute dose but the concentration in the clothes decreases by the time over the washing and last but not least there are many other sources of BT.

## Conclusions

The present study has investigated skin permeation from textiles with BT as model compound. Permeation studies were performed with both Franz-type and flow-through diffusion cells using an artificial skin mimicking model membrane, Strat-M®. Both methods show similar and consistent results and they demonstrate the ability of BT to migrate from textile materials to the skin surface and further penetrate to the deeper dermal layers with possible systemic exposure as a result. The Franz-type diffusion cells allowed BT recovery up to 80%, keeping the sink conditions throughout the experiment, due to the relatively high BT water solubility. The Franz cell system is however not suitable for less soluble compounds, since the amount of chemicals will be too low to be detected and quantified. The flow-through system gave lower recovery, between 59 and 71%, which is attributed to evaporation and/or adsorption of BT in the connecting tubing. However, flow-through system makes it possible to investigate hydrophobic compounds due to the larger amount of receiving solvent.

The results indicate that there are significant risks of transfer to, penetration of, and accumulation in human skin of BT. A deeper penetration makes further transport possible and thus giving rise to a systemic exposure. In previous studies, we identified several hazardous compounds in clothing obtained from the common market. Combined, this strongly indicates that there is a need for further studies focused on textile contaminants as they seem to be a forgotten route to human chemical exposure. It is also possible that this kind of studies can be used in carcinogenic and non-carcinogenic risk assessment of clothes in contact with the skin.

## Electronic supplementary material

ESM 1 (DOCX 44 kb)
