# Supplementary material for: Chemicals from textiles to skin: an in vitro permeation study of benzothiazole
Source: Environ Sci Pollut Res Int. 2018 Jun 17;25(25):24629–38. doi: 10.1007/s11356-018-2448-6 (PMC6133113; doi:10.1007/s11356-018-2448-6)
Supplement: Supplementary file 1 — (DOCX 44 kb) [file 11356_2018_2448_MOESM1_ESM.docx]

**Electronic Supplementary Material (ESM)**

**Chemicals from textiles to skin: an in vitro permeation study**

Francesco Iadaresta^1^, Michele Dario Manniello^2^, Conny Östman^1^*, Carlo Crescenzi^1,2^, Jan Holmbäck^1^*, Paola Russo^2^.

1. Department of Environmental Sciences and Analytical Chemistry (ACES), Stockholm University, Arrhenius Laboratory, Stockholm University SE-10691 Stockholm, Sweden
2. Department of Pharmacy, University of Salerno, Via Giovanni Paolo II, 132, I-84084 Fisciano, SA, Italy

*Corresponding authors:
Jan Holmbäck, [jan.holmback@aces.su.se](mailto:jan.holmback@aces.su.se)
Conny Östman, conny.ostman@aces.su.se

**TABLE OF CONTENTS**

**TABLE S1**: The mass spectrometer settings for BT and MeBT (IS).

**TABLE S2:** Benzothiazole concentration in clothes from literature data (G. Luongo, 2016), from where the median has been calculated.

**TABLE S3:** Literature data from where mean density for T-shirt has been collected (Rovira, 2015).

**TABLE S4:** Quality controls, precision and accuracy*.*

**TABLE S1**: Mass spectrometer settings for BT and MeBT (IS).

|  | **Quantification** | **Confirmation** | **Collision Energy** | **Declustering Potential** | **Entrance Potential** |
| --- | --- | --- | --- | --- | --- |
|  | **transition** | **transition** | **[eV]** | **[V]** | **[V]** |
| BT | 136 > 109 | 136 > 65 | 47 | 60 | 10 |
| MeBT (IS) | 150 > 109 | 150 > 65 | 34 | 13 | 10 |

**TABLE S2:** Benzothiazole concentration in clothes from literature data (G. Luongo, 2016), from where the median has been calculated.

| **Sample name** | **Color** | **Materials** | **Cloth Kind** | **Country** | **BT conc (ng/g)** |
| --- | --- | --- | --- | --- | --- |
| T-1 | Turquoise | 100 % Polyester | Female T-shirt | Cambodia | 1160 |
| T-2 | Jade green | 100 % Silk | Pillow case | India | 297 |
| T-3 | Black | 100 % Polyester | Sport trousers kid | China | 857 |
| T-4 | Blue | 100 % Modal | Scarf | China | 229 |
| T-5 | Violet | 90 % recycled polyamide, 10 % Elastane | Tights | Italy | 99.4 |
| T-6 | Blue | 88 % polyamide, 12 % Elastane | Tights | Turkey | 231 |
| T-7 | Beige | 80 % Polyamide, 20 % Elastane | Hotpants | Sri Lanka | 228 |
| T-8 | Black | 80 % Polyamide, 20 % Elastane | Hotpants | Sri Lanka | 212 |
| T-9 | Green | 87 % Polyester recycled, 13 % Elastane | Male T-shirt | China | 212 |
| T-10 | Green | 73 % Polyamide, 18 % Polyester, 6 % Elastodiene, 3 % Elastane | Socks | China | 866 |
| T-11 | Pink | Shell: 81 % Polyester, 19 % Elastane, Lining: 100 % Polyester | Swimsuit baby | Indonesia |  |
| T-12 | Pink | 100 % Recycled Polyester | Female T-shirt | Thailand | 789 |
| T-13 | Orange/Grey | 87 % Polyester, 13 % Elastane | Skirt | Indonesia | 459 |
| T-14 | Orange | 100 % Polyester | T-shirt | Thailand | 1590 |
| T-15 | Grey/Black | 88 % Polyester, 12 % Elastane | T-shirt | Unknown | 587 |
| T-16 | Pink | 95 % Polyester, 5 % Elastane | T-shirt | Unknown | 846 |
| T-17 | Black | 58 % Cotton, 39 % Polyester, 3 % Elastane | Trousers | Bangladesh | 447 |
| T-18 | Black | 100 % Polyester | Jacket | China | 348 |
| T-19 | Black | 97 % Cotton, 3 % Elastane | Trousers | Bangladesh | 133 |
| T-20 | Black | 67 % Viscose, 28 % Polyamide, 5 % Elastane | Trousers | China | 210 |
| T-21 | Yellow-green | 100 % Polyester | T-shirt kid | Indonesia | 0 |
| T-22 | Light blue | 85 % Polyester, 15 % Elastane | T-shirt kid | China | 0 |
| T-23 | Cobalt | 88 % Polyester, 12 % Elastane | Shorts | Vietnam | 741 |
| T-24 | Blue | 100 % Polyester | T-shirt kid | Unknown | 0 |
| T-25 | Fuchsia | 88 % Polyester, 12 % Elastane | Top | Sri Lanka | 440 |
| T-26 | Blue | 100 % Polyester | Shorts kid | China | 383 |
| T-27 | Blue | 100 % Polyester | Trousers | China | 831 |

**TABLE S3:** Literature data from where mean density for T-shirt has been collected (Rovira, 2015).

| N° | Cloth | Place | Materials | Made in | Colour | Density |
| --- | --- | --- | --- | --- | --- | --- |
| 1 | T-shirt | Sport store | 84% PE; 16% Sp | Sri Lanka | Black | *18* |
| 2 | Blouse | Chain store | 100% Vis | Turkey | Black | *9* |
| 3 | Underwear | Chain store | 95% Cot; 5% Sp | Portugal | Garnet | *22* |
| 4 | T-shirt | Chain store | 100% Cot | Bangladesh | Red | *14* |
| 5 | Baby Pyjama | Chain store | 100% Cot | Portugal | Pink | *19* |
| 6 | Bodysuit | Chain store | 100% Cot | China | Light blue | *25* |
| 7 | Baby Pyjama | Brand store | 80% Cot; 20% PE | Rumania | Light blue | *23* |
| 8 | Bodysuit | Brand store | 100% Cot | Tunisia | Pink | *24* |
| 9 | Underwear | Brand store | 92% Cot; 8% Sp | Bulgaria | Olive green | *21* |
| 10 | Blouse | Hypermarket | 100% Cot | India | Blue | *9* |
| 11 | Bodysuit | Hypermarket | 100% Cot | India | Light blue | *20* |
| 12 | Baby Pyjama | Hypermarket | 75% Cot; 25% PE | China | Light grey | *24* |
| 13 | Blouse | Hypermarket | 100% Cot | Bangladesh | Green | *14* |
| 14 | Underwear | Hypermarket | 92% PA; 8% Sp | Portugal | Black | *34* |
| 15 | T-shirt | Sport Store | 100% PE | China | Green | *19* |
| 16 | T-shirt | Sport Store | 100% PE | Tunisia | Blue | *16* |
| 17 | Blouse | Brand store | 68% Cot; 28% PA; 4% Sp | Bangladesh | White | *13* |
| 18 | Baby Pyjama | Brand store | 100% Cot | Bangladesh | White | *20* |
| 19 | Bodysuit | Brand store | 100% Cot | Bulgaria | White | *20* |
| 20 | Underwear | Brand store | 92% PA; 8% Sp | Spain | White | *26* |
| 21 | T-shirt | Sport Store | 100% PE | China | White | *17* |
| 22 | Blouse | Brand store | 100% Cot | India | White | *6* |
| 23 | T-shirt | Brand store | 100% Cot | Turkey | White | *15* |
| 24 | T-shirt | Hypermarket | 100% Cot | Bangladesh | White | *15* |
| 25 | Blouse | Hypermarket | 95% PE; 5% Sp | China | White | *20* |
| 26 | Underwear | Hypermarket | 100% Cot | Spain | White | *18* |
| 27 | Baby Pyjama | Hypermarket | 100% Cot | China | White | *17* |
| 28 | Bodysuit | Hypermarket | 100% Cot | China | White | *18* |
| 29 | Underwear | Brand store | 95% Cot; 5% Sp | N.S. | White | *17* |
| 30 | Baby Pyjama | Brand store | 100% Cot | Peru | White | *19* |
| 31 | Bodysuit | Brand store | 100% Cot | Turkey | White | *21* |
| PE: polyester; Sp: spandex; Vis: viscose; Cot: cotton; PA: polyamide; N.S.: not specified; density in mg/cm^2^. | | | | | | |

**LC/DAD method evaluation**

A benzothiazole standard stock solution of 1.2 mg/mL were prepared and diluted in ultrapure water. The linear range investigated was from 0.9 to 125 µg/mL, **Figure S1**.


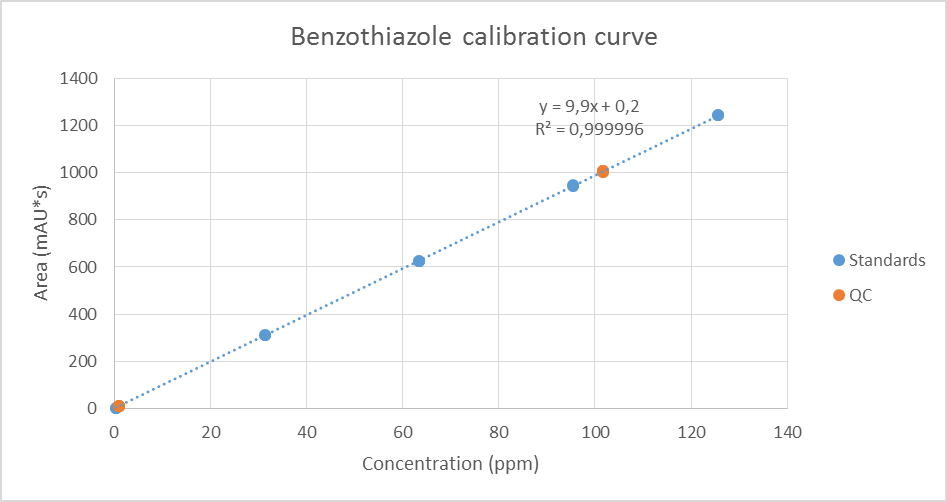


**Figure S1:** Calibration curve

The limit of detection (LOD) and limit of quantification (LOQ) were calculated using the standard deviation of noise divided by the slope times 3 and 10 respectively, where the standard deviation of noise was defined as the standard error of the calibration curve. The calculated concentration for LOD and LOQ were 0.28 and 0.95 µg/mL and instrumental LOD and LOQ of 1.4 and 4.7 ng injected. Quality controls were set at low and high concentration level. The low QC was approximatively the calculated LOQ (0.95 µg/mL) and the high QC was around the 80% of the last point in the calibration (101.6 µg/mL), in **Table S4** values for precision expressed as relative standard deviation and accuracy expressed as relative error.

**Table S4:**  Precision and accuracy for QC (n=5).

|  | **Concentration (µg/mL)** | **Precision (RSD %)** | **Accuracy (RE %)** |
| --- | --- | --- | --- |
| Low QC | 0.94 | 2.4 | -0.24 |
| High QC | 101.7 | 0.2 | 0.26 |
